# Tongxinluo promotes axonal plasticity and functional recovery after stroke

**DOI:** 10.1515/tnsci-2020-0127

**Published:** 2020-11-25

**Authors:** Xiaoting Wang, Xiaoqin Huang, Mengqi Yang, Xueying Pan, Meiyi Duan, Hui Cai, Guimiao Jiang, Xianlong Wen, Donghua Zou, Li Chen

**Affiliations:** Department of Neurology, Wuzhou Red Cross Hospital, Wuzhou, Guangxi Zhuang Autonomous Region, 543002, China; Department of Neurology, the First Affiliated Hospital of Guangxi Medical University , Nanning, Guangxi Zhuang Autonomous Region, 530021, China; Department of Neurology, the Fifth Affiliated Hospital of Guangxi Medical University , Nanning, Guangxi Zhuang Autonomous Region, 530021, China; Guangxi Key Laboratory of Regenerative Medicine and Guangxi Collaborative Innovation Center for Biomedicine, Guangxi Medical University, Nanning, Guangxi Zhuang Autonomous Region, 530021, China

**Keywords:** tongxinluo, distal middle cerebral artery occlusion, biotinylated dextran amines, axonal remodeling; functional recovery

## Abstract

**Background:**

The aim of this study was to investigate the neural plasticity in contralesional cortex and the effects of tongxinluo (TXL) in cerebral ischemic rats.

**Methodology:**

We used stroke-prone renovascular hypertensive (RHRSP) cerebral ischemia rat models to study the effect of TXL and the underlying mechanisms. We performed foot-fault and beam-walking tests to evaluate the motor function of rats after cortical infarction. Biotinylated dextran amine (BDA) was used to track axonal sprouting and neural connections.

**Results:**

TXL enhanced the recovery of motor function in cerebral infarction rats. TXL increased axonal sprouting in the peri-infarcted area but not in the corpus callosum, indicating in situ origination instead of crossing between cortical hemispheres through the corpus callosum. TXL promoted the sprouting of corticospinal axons into the denervated side of spinal gray matter. The synaptophysin (SYN)-positive intensity in the peri-infarcted area of TXL-treated group was greater than that in the vehicle group. We observed co-localization of SYN with BDA-positive fibers in the denervated spinal cord gray matter in the TXL group, suggesting that axonal remodeling and synaptic connections were promoted by TXL.

**Conclusion:**

TXL may promote the recovery of neurological function by promoting the axonal remodeling and synapse formation of motor neuronal fibers after focal cortical infarction in hypertensive rats.

## 1 Introduction

Stroke is a manifestation of ischemic injury of brain cells, which is often associated with hypertension in clinical settings. Stroke is a potentially fatal condition that also affects the quality of life of the afflicted individual [1,2]. We have adopted distal middle cerebral artery occlusion (dMCAO) [3] to induce ischemia in stroke-prone renovascular hypertensive (RHRSP) rats [4,5], to investigate the underlying mechanisms and explore potential treatment modalities.

In the dMCAO model, the infarct area is limited to the cerebral cortex and the subcortical nerve fibers are intact, which is an ideal setting for antegrade or retrograde studies involving the cortical spinal tract [3]. Previous studies have shown altered cortical expression of growth-related proteins contralateral to the infarcted cortex, and structural remodeling of the motor cortical nerve fibers in the contralateral spinal cord [6,7]; this phenomenon of spontaneous recovery may be involved in the repair of neurological deficit [8]. In clinical settings, most patients spontaneously recover to a certain extent after cerebral infarction [9,10], the mechanism of which is yet to be fully elucidated. Most of the studies till date have focused on the remodeling of peripheral nerve tissue in the infarcted site [11,12], and there is sufficient evidence to suggest partial involvement of this mechanism in neural functional recovery [13]. However, studies have also shown some limitations of spontaneous recovery including the over-large infarct size, scar tissue hyperplasia around the infarct, impaired vascular recanalization or angiogenesis, secondary damage to distal area, and the unstable neural stem cell activation and differentiation; all these factors limit the neurological functional recovery in patients [14]. Studies have shown that contralateral corticospinal tract (CST) axon sprouting and collateral formation occur earlier than that at the focal injury site during nerve injury remodeling in mature mammalian models, which also showed more complete functional repair [15]. When secondary cerebral infarction occurs in the contralateral cerebral motor cortex, the impairment of original ipsilateral limb movement is liable to be exaggerated, and the effect of treatment is far less than that before the cerebral infarction [16]. These findings collectively suggest that the contralateral brain is integrated into the function of the repair network system to promote recovery of limb motor function. Development of therapeutic agents for stroke associated with hypertension is a key imperative. Tongxinluo (TXL) is a Traditional Chinese Medicine remedy that is composed of extracts from 12 kinds of Chinese medicines in exact proportion. In previous studies, its use has been shown to be beneficial in various neurological diseases [17,18]. Administration of TXL 24 h after cortical infarction was shown to attenuate neuronal loss, promote neurogenesis and angiogenesis in the ipsilateral thalamus, and alleviate neurological impairment after focal cortical infarction in hypertensive rats [17]. Another study showed enhanced neurogenesis and angiogenesis in the peri-infarcted area of cortex and sub-ventricular zone, which may help to promote functional recovery after focal cerebral ischemia in hypertensive rats [19]. These results indicate a beneficial role of TXL in the recovery of neural function in the focal infarction area. However, no study has investigated the neural plasticity in contralesional cortex and the effects of TXL. Therefore, in the present study, we utilized biotinylated dextran amine (BDA) tracer to track the neural connections and investigated the neural plasticity in contralesional side, as well as the effect of TXL in hypertensive rats.

## 2 Materials and methods

### 2.1 Components of TXL capsule

TXL capsule used in this study was provided by Shijiazhuang Yiling Pharmaceutical Co., Ltd. Generally, TXL comprises of 12 components (Table 1). All components were authenticated and standardized according to the markers described in the Chinese Pharmacopoeia 2005 (National Pharmacopoeia Committee, 2005). In addition, the ingredients of TXL were strictly standardized as previously described [20,21]. TXL capsule was dissolved in distilled water to achieve a concentration of 3 mL/kg (0.5 g/kg/day), and the solution was intragastrically administered to rats at a dose of 500 mg/kg body weight daily for 28 days.

**Table 1:** Pharmaceutical ingredients of TXL

Components	Family	Voucher specimen number	Part used	Amount used (%)	Processing
Borneolum syntheticum insects	Dipterocarpaceae	11007	Resin	3.626	Artificial
*Panax ginseng* C.A. Mey.	Araliaceae	11001	Root and rhizome	1.677	Extraction
*Ziziphus jujuba* Mill. var. spinosa (Bunge) Hu ex H.F. Chou	Rhamnaceae	11002	Seed	1.173	Extraction
*Paeonia lactiflora* Pall.	Ranunculaceae	11003	Root	1.588	Extraction
*Santalum album* L.	Santalaceae	11004	Heartwood of stem	0.354	Extraction
*Dalbergia odorifera* T. Chen	Leguminosae	11005	Heartwood of stem and root	4.000	Extraction
*Boswellia carteri* Birdw	Burseraceae	11006	Resin	5.927	Farina
*Scolopendra subspinipes mutilans* L. Koch	Psittacidae	12001	Dried body	3.623	Farina
*Buthus martensii* Karsch	Buthidae	12002	Dried body	18.111	Farina
*Hirudo nipponica* Whitman	Hirudinidae	12004	Dried body	27.330	Farina
*Cryptotympana pustulata* Fabricius	Cicadidae	12005	Skin	18.111	Farina
*Steleophaga plancyi* (Boleny)	Corydiidae	12003	Female dried body	18.111	Micro-oryzae farina

### 2.2 Animals

**Figure 1:**
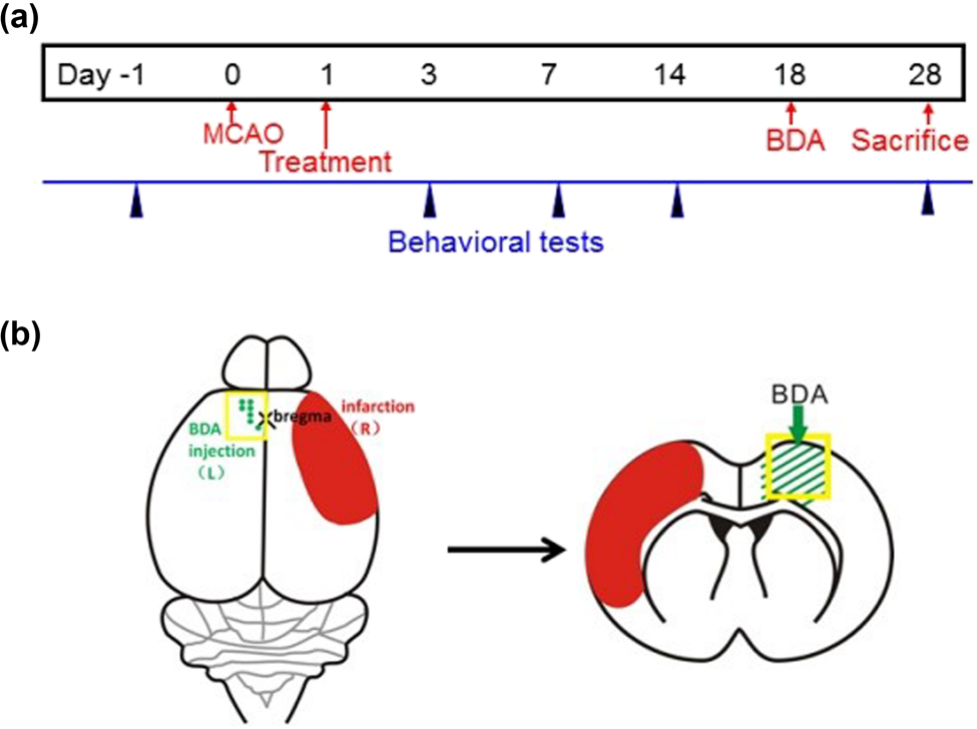
The experimental design and overview of the cerebral ischemia model. (a) Time line of the experiments. The time-points for pre-training of rats, dMCAO surgery, TXL or vehicle application, BDA injection, and sacrifice of rats are shown in red. Blue pointers indicate the time points for behavioral tests. (b) Schematic diagrams showing the locations of BDA injection and ischemic area in transverse section (left panel) and coronal section (right panel). Abbreviations: dMCAO, distal middle cerebral artery occlusion; BDA, biotinylated dextran amines.

The experimental design and overview of the cerebral ischemia model are shown in Figure 1.

**Ethical approval:** All animal experiments were approved by the Animal Research Ethics of the First Affiliated Hospital of Guangxi Medical University (No. 2019 [KY-E-044]). The use of animals in the experiments had observed the Interdisciplinary Principles and Guidelines. All possible efforts were made to minimize the number of animals used in this research and their suffering. Rats were not euthanized with chloral hydrate.

### 2.3 Stroke-prone RHRSP rat model

To establish stroke-prone RHRSP rats, 24 male Sprague Dawley rats weighing 70–90 g and aged 4 weeks were used according to the method described previously [5]. Rats were kept in the animal center of The First Affiliated Hospital of Sun Yat-sen University in 22–24°C environment with free access to food and water with 12 h dark and light cycles.

Briefly, rats were anesthetized with 10% chloral hydrate by intraperitoneal injection (350 mg/kg); there was no discomfort such as peritonitis and pain in rats after intraperitoneal injection of 10% chloral hydrate and then operated to induce bilateral renal artery constriction. Systolic blood pressure was monitored once weekly using a tail-cuff sphygmomanometer. Twelve weeks after RHRSP, 20 rats that had systolic blood pressure consistently higher than 180 mmHg and that exhibited no stroke symptoms were assigned to receive permanent dMCAO operation as previously described [22]. Briefly, rats were anesthetized with 10% chloral hydrate [350 mg/kg, intraperitoneally (i.p.)], and then the right middle cerebral artery was occluded distal to the origin of the striatal branches by micro-bipolar coagulation, leading to the permanent focal infarction in the neocortex. After 24 h, fulfilling the following two criteria will be taken as a successful dMCAO surgery: left forelimb flexion upon being lifted by the tail and circling to the left side when crawling forward. The 16 rats with successful dMCAO were randomly divided into two groups: vehicle and TXL groups (*n* = 8 per group). Four rats were excluded from this study because of failed dMCAO, intracranial hemorrhage, or death during the experiment. Rats were killed 28 days after dMCAO.

### 2.4 Foot-fault test

The foot-fault test was used to evaluate fine motor function of the affected limb. A 110 cm long, 10 cm wide rectangle grid consisted of several wires (1 mm in diameter) with grid cells measuring 3 cm × 3 cm was placed 1 m above the ground [23]. Rats were trained to pass completely through the grid in 1 min, thrice a day for five consecutive days before dMCAO. The affected limb falling off or slipping through the grid was counted as a foot fault. The foot-fault rate of the affected limb was determined by the ratio of the number of fault footsteps to the total number of steps. The test was repeated thrice for each rat by an evaluator blinded to the group division and was performed before dMCAO surgery, as well as 3, 7, 14, and 28 days after surgery. The average foot-fault rate of three tests at each time point was calculated for each rat.

### 2.5 Beam-walking test

Fine motor coordination was evaluated by beam-walking test. A 120 cm long, 25 mm wide wooden beam placed 1 m above the ground was used in the test [24]. Rats were trained to walk on the crossbar thrice a day for five consecutive days before dMCAO. After dMCAO, beam-walking performance was rated according to the following criteria: 0, unable to stay on the beam and fall; 1 point, able to stay but unable to walk through the beam; 2 points, fall when walking through the beam; 3 points, traversed the beam without using the disabled limb; 4 points, traversed the beam using the disabled limb in less than 50% of the steps; 5 points, traversed the beam using the disabled limb in more than 50% of the steps; 6 points: traversed through the beam normally. The test was performed 1 day before dMCAO surgery, as well as at 3, 7, 14, and 28 days after surgery. The average score of three tests at each time point was calculated for each rat.

### 2.6 BDA tracking and analysis

On the eighteenth day after dMCAO surgery, rats were injected with 1.4 µL of the anterograde axonal tracer BDA (molecular weight 10,000 Da; Molecular Probes, Oregon, USA) into the left motor cortex (contralateral to the lesion side) according to the modified method [25]. Briefly, after being anesthetized with 10% chloral hydrate solution (350 mg/kg, i.p.), the rats were placed in a stereotactic device. A small bone window with a length of 7 mm (from AP 4 to −3 mm) and a width of 4 mm (mediolateral [ML] 4 mm) was drilled on the skull overlying the left cerebral cortex. Ten percent BDA solution in 0.01 mol/L PBS (0.1 mg/mL) was microinjected into coordinates (anteropsterior [AP]: 3 mm, ML: 3 mm, dorsoventral [DV]: −2 mm; AP: 3 mm, ML: 2 mm, DV: −2 mm; AP: 2 mm, ML: 3 mm, DV: −2 mm; AP: 2 mm, ML: 2 mm, DV: −2 mm; AP: 1 mm, ML: 2 mm, DV: −2 mm; AP: 0 mm, ML: 2 mm, DV: −2 mm; AP: −1 mm, ML: 1 mm, DV: −2 mm) at a rate of 0.1 µL/min (0.2 µL for each coordinate), and the syringe was left in place for 3 min after each injection.

### 2.7 Tissue preparation

Twenty-eight days after dMCAO, rats were killed under deep anesthesia with 10% chloral hydrate (580 mg/kg) and transcardially perfused with saline, followed by 4% paraformaldehyde (Sigma-Aldrich) in phosphate buffer. Brains and cervical spinal cords were removed and postfixed in the same fixative for 24 h at 4°C, immersed sequentially in graded sucrose from 20 to 30% until they sank, and then frozen and embedded in opti-mum cutting temperature (OCT). Serial coronal sections (10-µm thick) were cut on a cryostat (CM 1900; Leica, Germany) for immunohistochemistry.

Nine cerebral sections for measurement of the average density of BDA-positive fibers in the ipsilesional M1 (primary motor cortex) and the lesion (from bregma 3.0 to −1 mm) of each rat were selected at regular intervals (0.5 mm). Six of these sections (from bregma 1.5 to −1 mm) for measurement of the average density of BDA-positive fibers in the middle portion of the corpus callosum and four sections for measurement of the average synaptophysin (SYN) fluorescence intensity were also prepared. Furthermore, 25 equally spaced sections from the upper cervical spinal cord were randomly selected for counting the number of BDA-positive fibers. Among these, some spinal sections were used to determine the relationship between the BDA-positive fibers and the SYN protein through immunofluorescence co-localization analysis.

### 2.8 Infarct volume measurement

Nissl staining with 0.1% cresyl violet (Sigma-Aldrich) was performed to measure infarct volume after dMCAO. The relative infarct volume was calculated as the percentage of lesioned volume relative to the unlesioned volume in the contralateral hemisphere.

### 2.9 Immunohistochemistry

A series of coronal sections were selected to perform immunohistochemistry analyses using standard procedures. Mouse monoclonal anti-SYN (1:200, Sigma, USA) was used as the primary antibody. After overnight incubation with primary antibody at 4°C, the fluorescent-labeled secondary antibodies Alexa Fluor 594-conjugated donkey anti-mouse (1:500; Invitrogen, USA) and Alexa 488-conjugated streptavidin (1:500; Invitrogen) were applied for 1 h at room temperature. Negative control sections were incubated with 0.01 M phosphate-buffered saline as a substitute for the primary antibody. Nuclei were counterstained with 4′,6-diamidino-2-phenylindole (DAPI 1:1,000; Sigma), and samples were mounted in ProLong Gold anti-fade solution (Molecular Probes). Fluorescence signals were detected using a microscope (Olympus BX51; Olympus).

### 2.10 Data analysis and statistics

#### 2.10.1 Brain tissue

Image-Pro-Plus software (IPP6.0; Silver Spring, USA) was used to measure BDA-positive axonal density. For each rat, a region of interest (315 µm × 315 µm) in the peri-infarcted cortex from each of the nine brain sections was randomly selected, as well as in the contralateral homologous cortex, and then the average density in both cortex was calculated for each animal. To avoid inter-animal variation in the tracing efficiency by a subtle difference of injection volume, a quotient coefficient was calculated through the individual average BDA-positive axonal density divided by the total mean density of all animals in the contralateral homologous cortex. The standardized BDA-positive axonal density for each individual animal was the product of the average BDA-positive axonal density and the quotient coefficient. The same measurement was also for the standardized BDA-positive axonal density in the corpus callosum within regions of interest (420 µm × 425 µm) [26].

To confirm whether TXL treatment changes the expressions of synaptic markers, three non-overlapping immunofluorescence images were captured at 10 × 40 magnification for the synaptic markers – SYN from the peri-infarcted cortex. The pixel of integrated density from four sections per animal was measured using IPP, and the average pixel was calculated.

#### 2.10.2 Cervical spinal cord tissue

BDA-positive fiber was shown in green with cord-like shape, different lengths, and multiple green branches. (1) For each of the selected consecutive 25 sections in each rat, the central canal was considered as the center dot with a radius of 300 or 600 μm, so that two cycles were set in the denervated side of the gray matter (i.e., ipsilateral to the BDA injection), which were then divided into three assuming regions A, B, and C. (2) For each rat, the average number of BDA-labeled CST in the contralesional dorsal funiculus at the C1 level was counted on five sections, and the total number of BDA-positive fibers in the stroke-impaired side of the gray matter was counted in the three assuming regions A, B, and C, respectively, on 25 consecutive sections under the field of 10 × 10 times. (3) To avoid inter-animal variation induced by tracing efficiency, the number of BDA-positive fibers in each rat was corrected with a quotient of BDA-positive fibers’ number in each area divided by the mean BDA-positive CST number in the dorsal funiculus at the C1 level.

#### 2.10.3 Data analysis

All data are presented as mean and standard deviation. Student’s *t*-test or one-way ANOVA was used to conduct the comparison between groups if the data were normally distributed. ANOVA was followed by Tukey *post hoc* tests where interactions were found if the data were normally distributed. Rank sum test was performed if the data were not normally distributed and indicated as M. Statistical analyses were performed using SPSS 17.0 (IBM, USA). *P* < 0.05 was considered indicative of significant difference.

## 3 Results

### 3.1 TXL enhanced neurological functional recovery after focal cortical infarction in hypertensive rats

**Figure 2:**
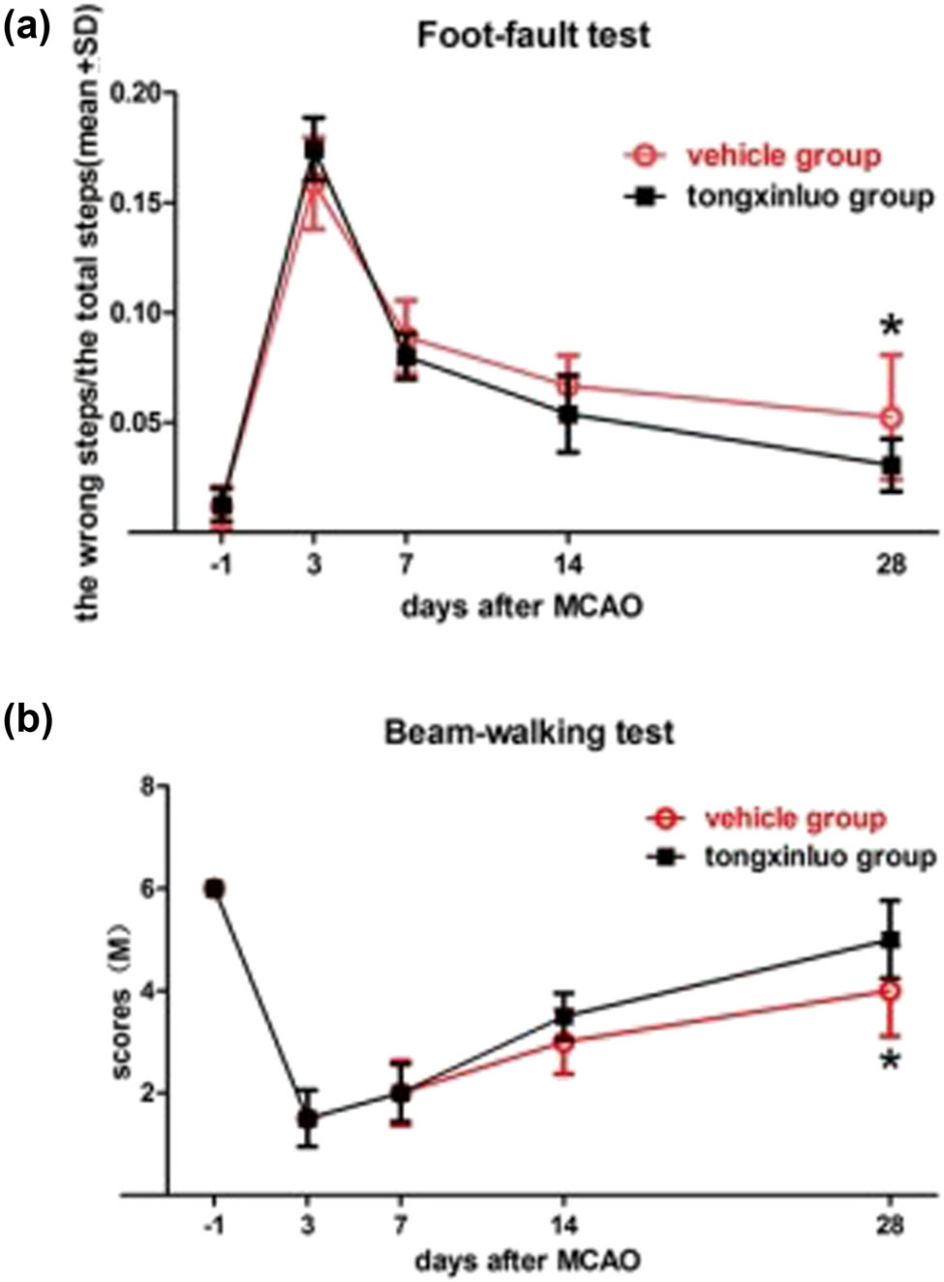
Results of the foot-fault and beam-walking behavioral tests. (a) Foot-fault test: percentage of wrong footsteps (out of total footsteps) on days −1, 3, 7, 14, and 28 after dMCAO in the vehicle and TXL groups. On postoperative day 28, the wrong footstep rate of TXL groups (0.0305 ± 0.012) was lower than that of vehicle groups (0.0523 ± 0.0284). (b) Beam-walking test: the score of muscle strength degree on days −1, 3, 7, 14, and 28 after dMCAO in the vehicle and TXL groups. On postoperative day 28, the scores of TXL groups (5 ± 0.76) were higher than that of vehicle groups (4 ± 0.89). Data expressed as mean ± standard deviation. *P < 0.05 on ANOVA Tukey *post hoc* test. *n* = 8 rats per group.

Lesion size varied among rats in each group; however, there was no significant difference between the vehicle and TXL treatment groups (lesion size = 114.4 ± 10.2 mm^3^ [mean ± SEM] in the saline-treated group and 120.1 ± 12.6 mm^3^ in the TXL-treated group).The neurological function was tested by the foot-fault test and beam-walking test. All rats subjected to dMCAO exhibited a progressive recovery over time (Figure 2). However, TXL-treated rats showed lower wrong footstep rate in the foot-fault test (Figure 2a, *P* < 0.05) and better scores in the beam-walking test (Figure 2b, *P* < 0.05) 28 days after dMCAO.

### 3.2 TXL increased axonal sprouting in the ipsilesional cortex

**Figure 3:**
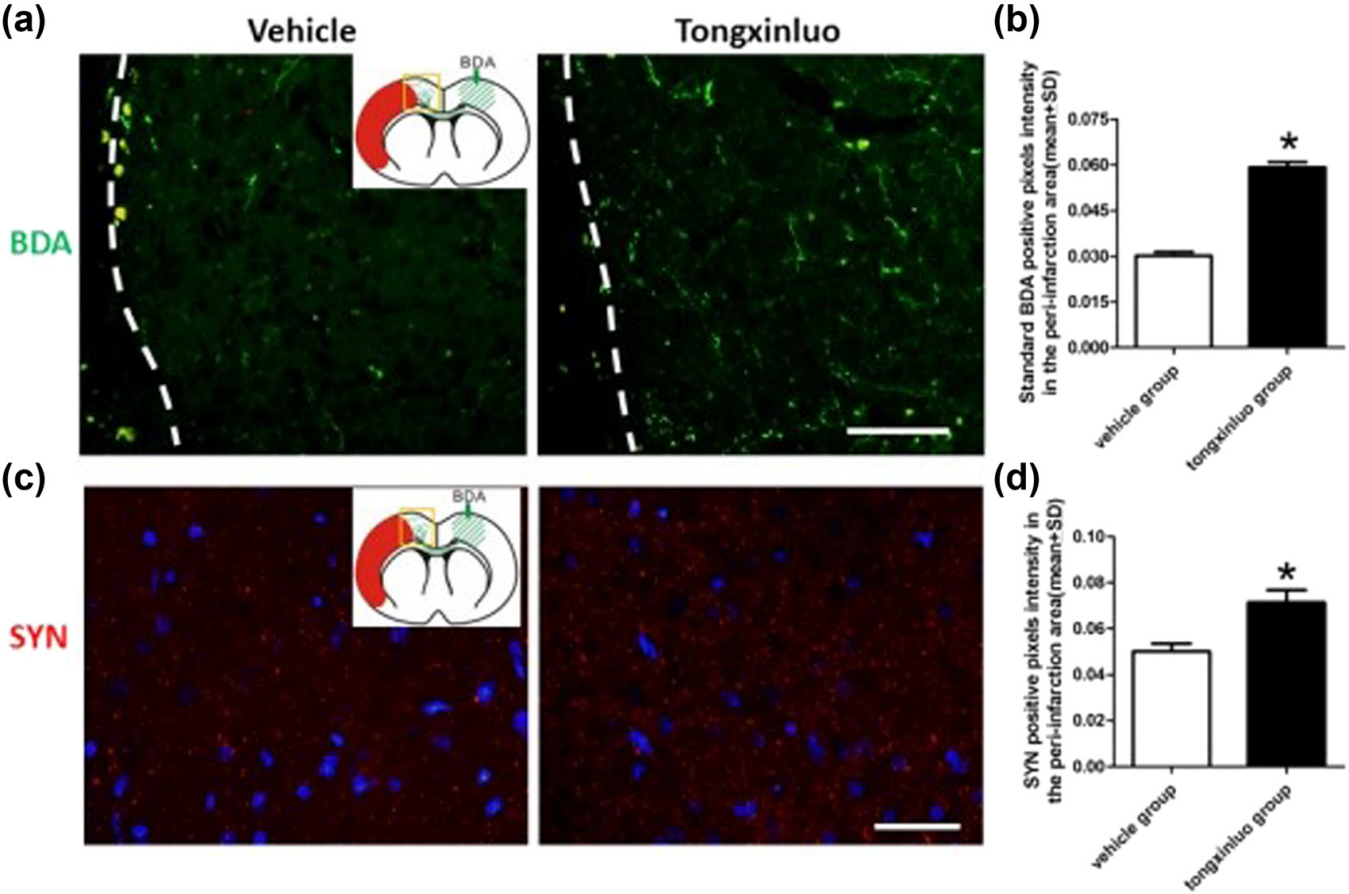
Axonal and SYN labeling in the peri-infarcted area. (a) Representative images showing the BDA-labeled axons in the peri-infarcted area in the vehicle and TXL groups. Scale bar = 100 μm. (b) Compared with the vehicle groups (0.0302 ± 0.0037), TXL significantly increased the density of BDA-labeled fibers (0.0591 ± 0.0055) in the peri-infarcted area at 28 days after dMCAO (*P < 0.05). (c) Representative images of SYN and DAPI merge in the peri-infarcted area in vehicle and TXL groups. Scale bar = 40 μm. (d) Compared with the vehicle groups (0.05 ± 0.0165), TXL significantly increased SYN expression (0.0714 ± 0.0264) in the peri-infarcted area at 28 days after dMCAO (*P < 0.05). *n* = 10 samples per group. Red mass = infarcted area; Green arrow = BDA injection site; yellow square = sample site.

**Figure 4:**
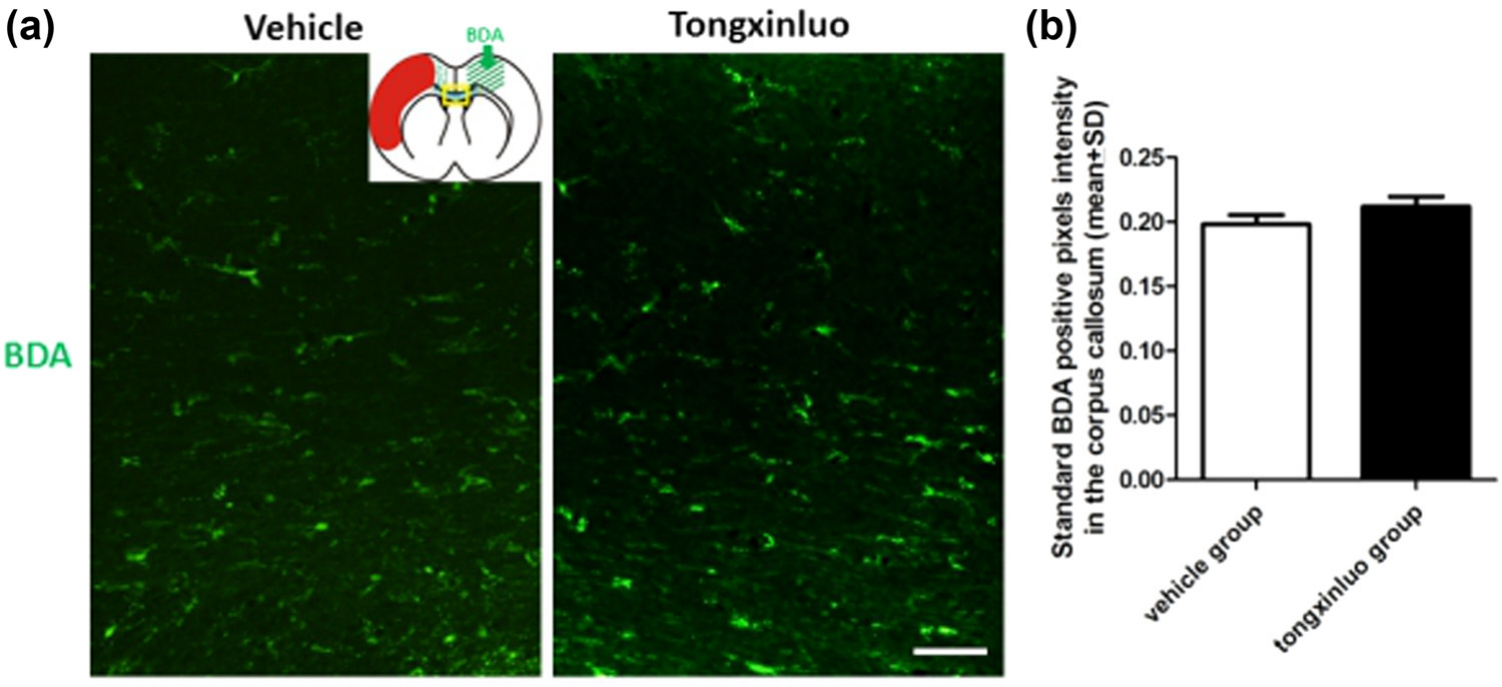
Axonal labeling in the corpus callosum. (a) Representative images showing the transcallosal axons in the middle portion of corpus callosum in the vehicle and TXL groups. (b) There are no significant differences with respect to axonal quantification in the corpus callosum between rats treated with vehicle (0.1983 ± 0.0222) or TXL (0.2121 ± 0.0243) (*P > 0.05). Scale bar = 50 μm. *n* = 10 samples per group. Red mass = infarcted area; green arrow = BDA injection site; yellow square = sample site.

To determine whether neurons in contralesional cortex undergo axonal sprouting toward the homologous area in the ipsilesional cortex, we injected the anterograde axonal tracer BDA into the contralesional cortex as shown in the schematic diagram. TXL-treated rats exhibited significantly higher axonal density in the ipsilesional cortex as compared to that in vehicle-treated animals at 28 days after dMCAO (Figure 3a and b). To further substantiate TXL-induced plasticity, we found that TXL treatment significantly enhanced the expression of presynaptic marker SYN in the ipsilesional cortex (Figure 3c and d). To determine whether increased axons in the ipsilesional cortex reflect post-stroke axonal regeneration originating from the contralesional cortex, we measured BDA-positive axonal density in the area crossing the midline of the corpus callosum on coronal sections of the vehicle control and TXL-treated rats. Our data showed that axonal density was essentially unaltered by either vehicle or TXL treatment (Figure 4). This observation ruled out the possibility of new axonal generation along the corpus callosum between cortical hemispheres.

### 3.3 TXL promoted the sprouting of corticospinal axons crossing over the midline into the denervated side of spinal gray matter

**Figure 5:**
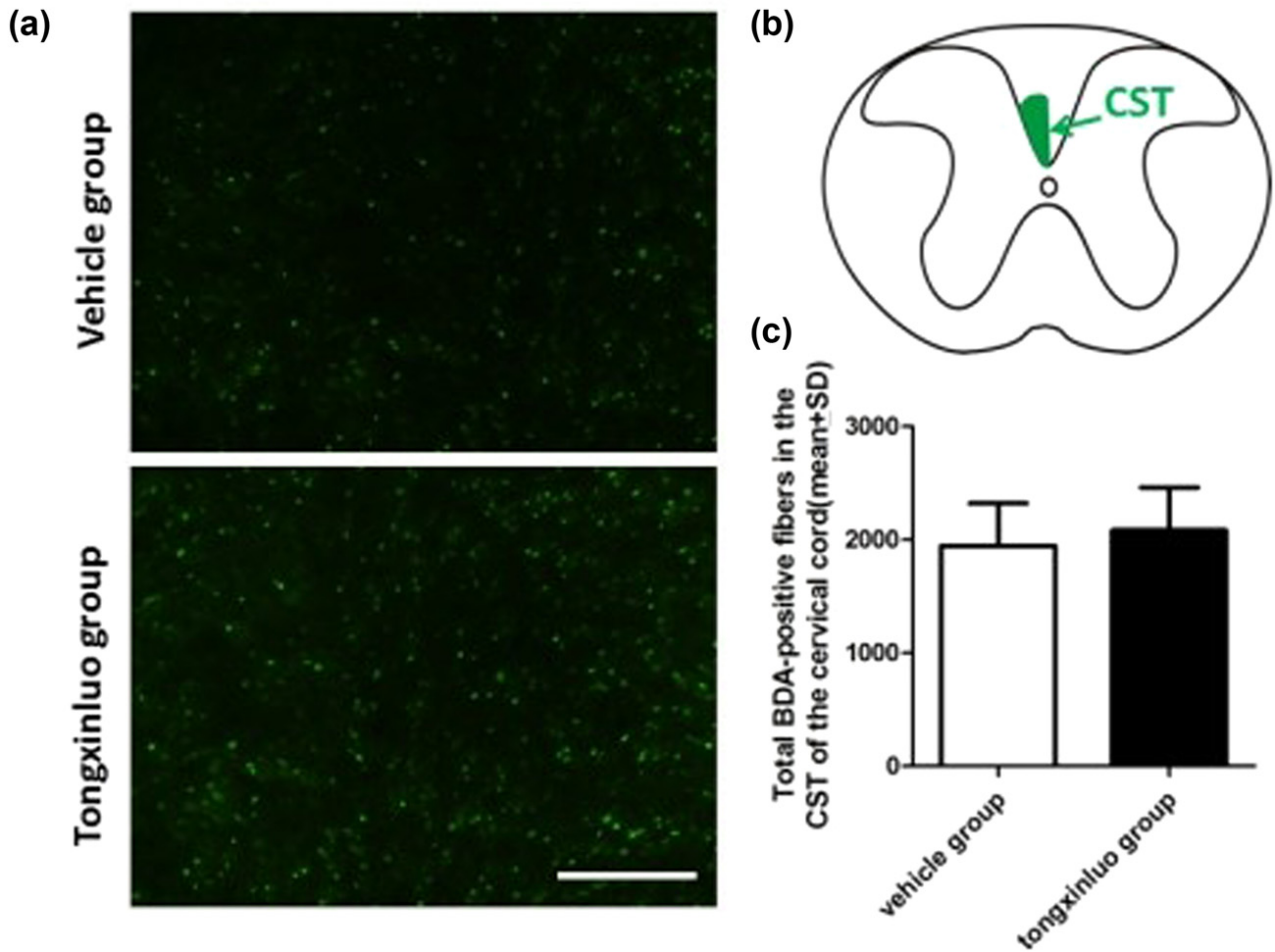
Axonal labeling of the CST at the cervical level. (a) Representative images showing the axons of the CST at the cervical level in vehicle and TXL groups. (b) Schematic diagram showing the sample site of the CST. CC: corpus callosum. Scale bar = 150 µm. (c) There are no significant differences with respect to CST axonal density quantification between rats treated with vehicle (1945.63 ± 374.85) or TXL (2084.25 ± 377.55) (*P > 0.05). Scale bar = 50 μm. *n* = 8 rats per group. Data expressed as mean ± standard deviation. *P < 0.05 on ANOVA Tukey *post hoc* test. ^#^ *P* < 0.05 on ANOVA Tukey *post hoc* test.

**Figure 6:**
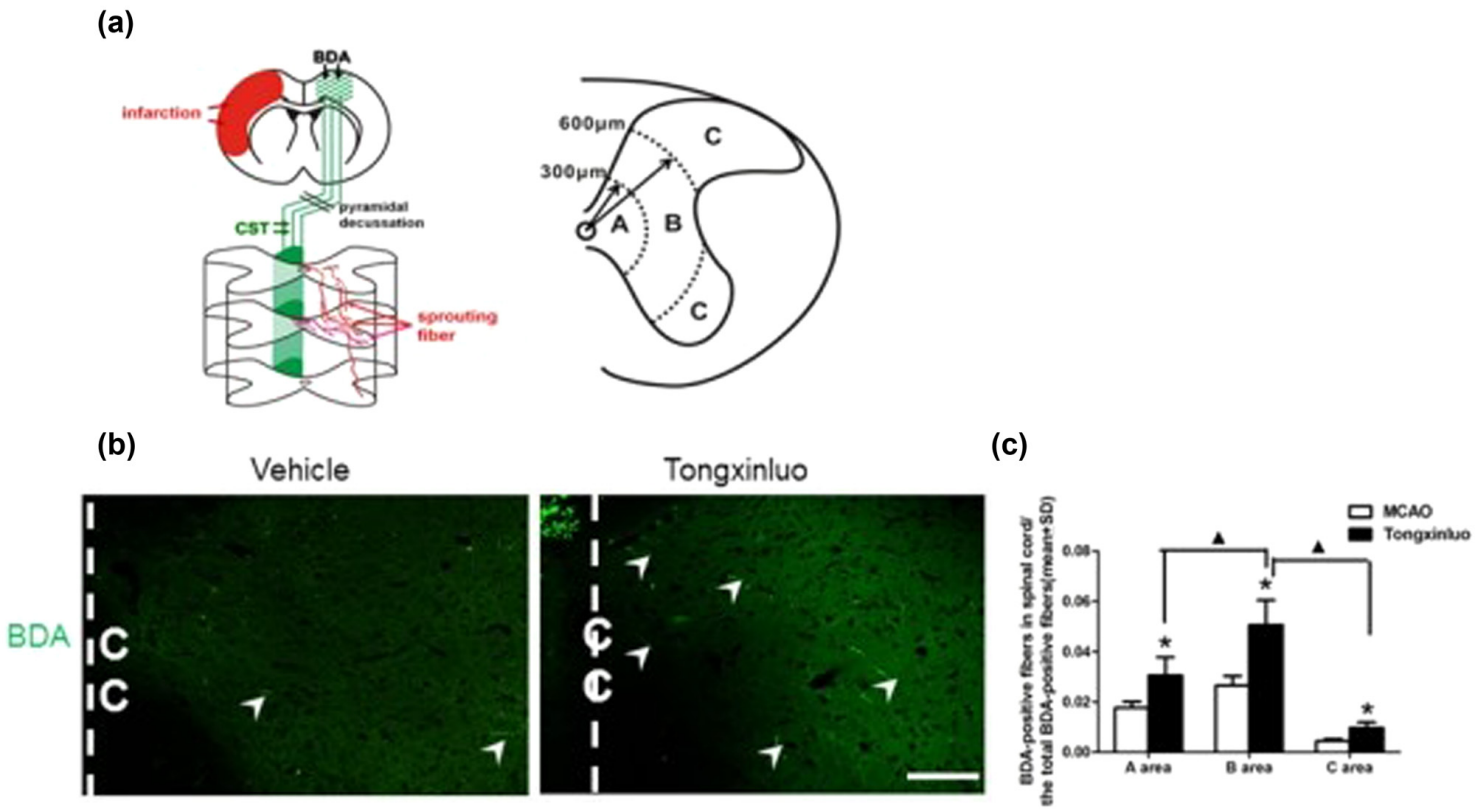
Sprouting axons in the denervated spinal cord at the cervical level. (a) Schematic diagrams showing the axons sprouting across midline at the cervical level. (b) Representative images showing the axons in the denervated spinal cord at the cervical level in vehicle and TXL groups. (c) TXL significantly increased the number of lengthy axons within the gray matter in the denervated spinal cord at the cervical level after dMCAO (*P < 0.05). Scale bar = 50 μm. *n* = 8 rats per group. Vehicle groups: quotient of BDA-positive fibers’ number in A area (0.0176 ± 0.0025), B area (0.0265 ± 0.0038), and C area (0.00446 ± 0.0009). TXL groups: quotient of BDA-positive fibers’ number in A area (0.0305 ± 0.0073), B area (0.0506 ± 0.0098), and C area (0.0096 ± 0.0023).

At the cervical level, the descending corticospinal fibers form neural circuits from spinal interneurons to spinal motoneurons at the same side of gray matter for the control of forelimb movement. To determine whether the neural pathway originating from the contralesional intact motor cortex reinnervate the denervated side of the spinal cord, we measured BDA-positive CST axonal density at the cervical level. The results showed no significant differences in CST axonal density quantification between rats with vehicle or TXL treatment (Figure 5a and c). However, axonal sprouting from the intact CST was observed in the denervated side of the gray matter in the vehicle control rats (Figure 6b). Furthermore, the CST sprouting was remarkably enhanced in the TXL-treated rats (Figure 6b). Moreover, TXL increased the number of lengthy axons within the gray matter in the denervated spinal cord (Figure 6c, *P* < 0.05). Our data indicate that TXL treatment significantly promoted axonal reorganization in the denervated spinal cord at the cervical level after dMCAO.

### 3.4 TXL treatment enhanced synapse formation on dendrites of motoneurons in the denervated side of the spinal cord

**Figure 7:**
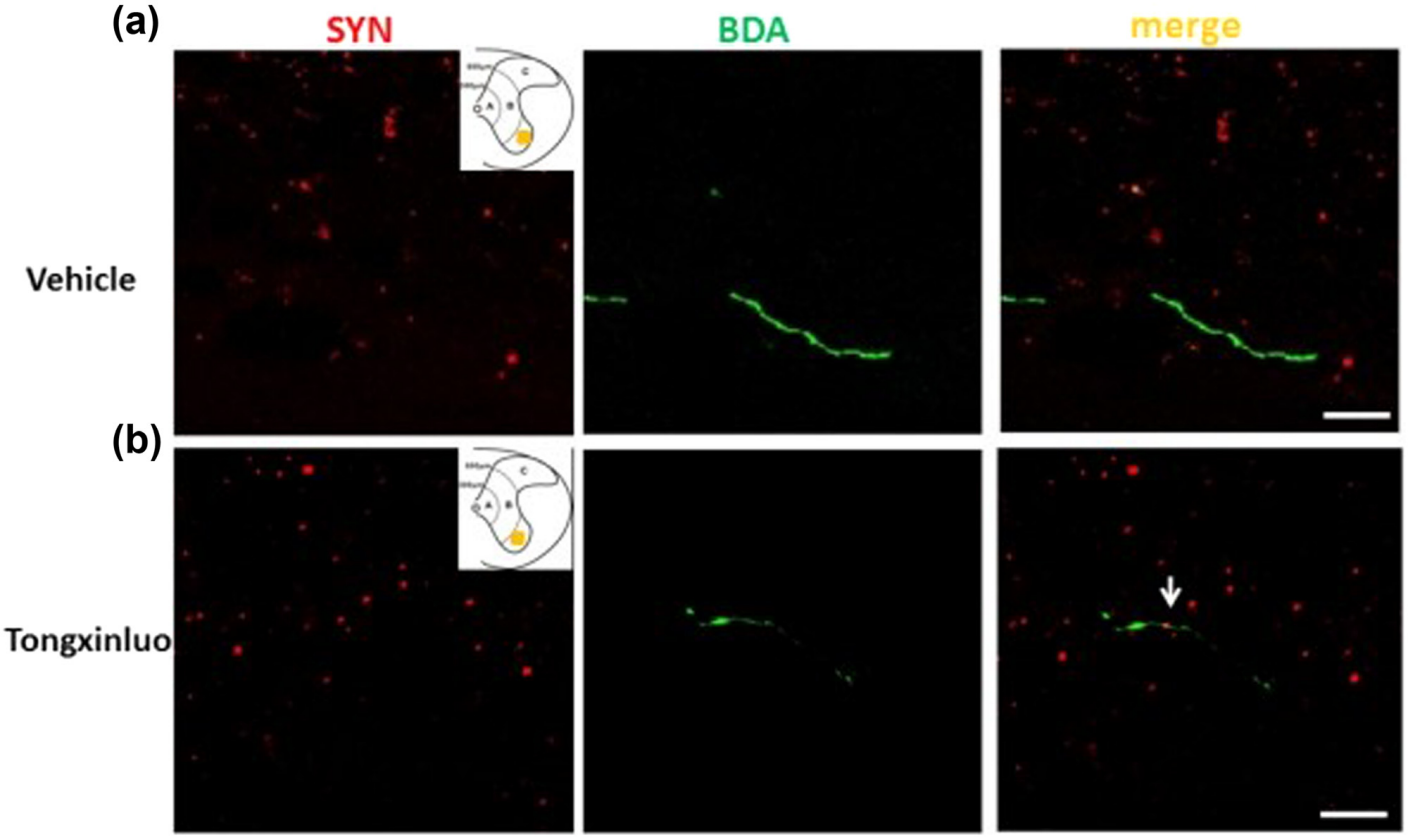
Spouting axons and synapse formation in the denervated spinal cord at the cervical level. (a) Representative image showing SYN proteins yielded punctated signals, which scattered along BDA-labeled dendrites of spinal motoneurons in the vehicle rats. (b) Representative image showing substantial SYN closely encircling the dendrites in the denervated spinal cord of TXL-treated rats. Scale bar = 10 μm. Yellow square = sample site.

To determine whether synaptic structure of denervated motoneurons underwent remodeling after TXL treatment, we further examined the expression of the presynaptic marker SYN at the cervical level. Immunostaining for the SYN proteins yielded punctate signals in the vehicle control rats, which scattered along BDA-labeled dendrites of spinal motoneurons (Figure 7a). However, substantial SYN closely encircling the dendrites was observed in the denervated side of the spinal cord in TXL-treated rats (Figure 7b). Our data indicate that TXL treatment significantly promoted synapse formation in the denervated spinal cord at the cervical level after dMCAO.

## 4 Discussion

The present study investigated the effect of TXL capsule on post-ischemic axonal plasticity after dMCAO surgery in a rat model of stroke-prone RHRSP. We found that TXL enhanced the recovery of motor function and muscle strength after ischemic injury as assessed by the foot-fault and beam-walking behavioral tests. Moreover, we used BDAs for tracking the neural connections; our results showed that TXL promoted BDA-positive fluorescence intensity in both the peri-infarcted area and the denervated spinal cord gray matter. In addition, we also found that TXL promoted SYN-positive intensity in the peri-infarcted area, as well as the co-localization of SYN with BDA-positive intensity in the denervated spinal cord gray matter. These findings suggest that new axonal growth and synaptic connections were promoted by TXL, and that these were functionally working. Collectively, the above results suggest that TXL can promote the recovery of neural function and connections after ischemic injury induced by dMCAO. Our results may provide a candidate target for the treatment of ischemia in hypertensive patients, as well as help explore the underlying mechanisms involved in protection of neural function from ischemic damage.

Hypertension is a common risk factor for cerebrovascular disease, and the dMCAO hypertensive animal model is believed to closely simulate the clinical outcomes as far as possible [27,28]. The blood pressure level in renal secondary hypertension model exhibited a steady increase, which simulates the progress of blood pressure in clinical hypertension, and can guarantee the stability of blood pressure without individual differences and genetic diversity. These attributes render dMCAO in RHRSP rats as a good model for stroke in hypertension [29,30].

Motor cortex is known to contain neurons that project directly to the spinal cord and activate somatic motor neurons to control the limb muscle movement [31,32]. Motor cortex was infarcted in the present dMCAO study; we showed that dMCAO surgery successfully impaired the performance of rats in the foot-fault and balance beam-walking tests, which is consistent with the results of previous studies [33,34]. In addition, application of TXL promoted motor function and muscle strength, which were impaired by dMCAO in hypertensive rats; this suggests a neuroprotective effect of TXL. We hypothesized that the TXL enhances the motor function and muscle strength via promotion of neural function and connections in nerve fibers’ projections.

The neuronal impairment and loss associated with focal brain infarcts are well documented. A previous study used the neuronal markers BrdU, nestin, and NeuN to show that TXL promotes neurogenesis and angiogenesis [17,19]. In this study, we first used BDA as a neuronal tracer to investigate the neural function and axonal reconstruction in the focal peri-infarcted area and in the denervated side of the spinal cord gray matter. We observed BDA-positive fluorescence in the focal peri-infarcted area, which was enhanced by TXL treatment; this indicates that TXL promotes axonal growth after dMCAO. In addition, no significant between-group difference with respect to BDA-positive nerve fibers was observed in the corpus callosum, whereas a significant difference was observed in the peri-infarcted area. Therefore, TXL can significantly promote the growth of axonal branches in the peri-infarcted cortex area, which is considered as one of the mechanisms of recovery of motor function. In addition, we found that TXL promoted SYN-positive intensity in the peri-infarcted area, as well as the co-localization of SYN with BDA-positive intensity in the denervated side of the spinal cord gray matter. These findings suggest that new axonal growth and synaptic connections were promoted by TXL, and that these were functionally working. The new axonal growth was produced by branches of nerve fibers in the peri-infarcted area but not from the contralesional side across corpus callosum. Similarly, the total number of BDA-positive fibers in the C1 area of CST was comparable between the two groups, which suggested that the new fibers were coming from the new axonal branches produced downstream of CST of contralesional side and crossed the central line of the spinal cord center, but not from the cortex to spinal cord. The TXL group exhibited significant promotion of new axonal growth in the C1 area downstream of the denervated spinal cord gray matter originated from the contralesional CST, reinnervating the denervated side of the spinal cord, which may be one of the mechanisms of motor functional improvement. The contralateral CST can spontaneously project to the denervated cervical spinal cord through the midline after focal infarction induced by dMCAO surgery [8,35,36,37], and some studies have examined the length of the nerve fibers [25]. Herein, to study the space distribution of the BDA-positive nerve fibers, we divided the denervated gray matter into three parts (A, B, and C areas); the present study is the first to report that the TXL group had more BDA-positive nerve fibers in all three areas. We noticed more BDA-positive nerve fibers in area B as compared to that in areas A or C; we speculate that these were coming from area A in the same layer and from areas A and B in the upper layer. In addition, the lower density of BDA-positive fibers observed in area C may be attributable to the long distance from A and B areas, which requires longer time to produce. However, the neural information exchange to regulate the locomotion function was occurring in area C, because it is rich in neurons owing to the presence of anterior horn of the spinal cord in this area. Therefore, we analyzed the co-localization of SYN and BDA in the C area. Our data indicate that TXL treatment significantly promoted synapse formation in the denervated spinal cord at the cervical level after dMCAO, reshaping the local motor microcircuit.

Collectively, our results indicate that TXL may promote the recovery of motor function and muscle strength by enhancing the lateral branch growth of motor neuronal fibers, and it may also promote the axonal remodeling and synaptic connections of motor neuronal fibers after focal cortical infarction in hypertensive rats. Our results provide a candidate target for the treatment of ischemic neuronal injury in hypertensive patients.
