# Corrigendum to “Tongxinluo promotes axonal plasticity and functional recovery after stroke”

**DOI:** 10.1515/tnsci-2022-0988

**Published:** 2024-12-21

**Authors:** Xiaoting Wang, Xiaoqin Huang, Mengqi Yang, Xueying Pan, Meiyi Duan, Hui Cai, Guimiao Jiang, Xianlong Wen, Donghua Zou, Li Chen

**Affiliations:** Department of Neurology, Wuzhou Red Cross Hospital, Guangxi Zhuang Autonomous Region, Wuzhou, 543002, China; Department of Neurology, The First Affiliated Hospital of Guangxi Medical University, Guangxi Zhuang Autonomous Region, Nanning, 530021, China; Department of Neurology, The Fifth Affiliated Hospital of Guangxi Medical University, Guangxi Zhuang Autonomous Region, Nanning, 530021, China; Guangxi Key Laboratory of Regenerative Medicine and Guangxi Collaborative Innovation Center for Biomedicine, Guangxi Medical University, Guangxi Zhuang Autonomous Region, Nanning, 530021, China

Corrigendum to Wang X, Huang X, Yang M, Pan X, Duan M, Cai H, Jiang G, Wen X, Zou D, Chen L. Tongxinluo promotes axonal plasticity and functional recovery after stroke. Translational Neuroscience. 2020;11(1):428–438. https://doi.org/10.1515/tnsci-2020-0127.

After the publication of this article, the authors identified an error and subsequently made the necessary correction.

**Erroneous figures:**

This is the misused Figure 5 in the published article.

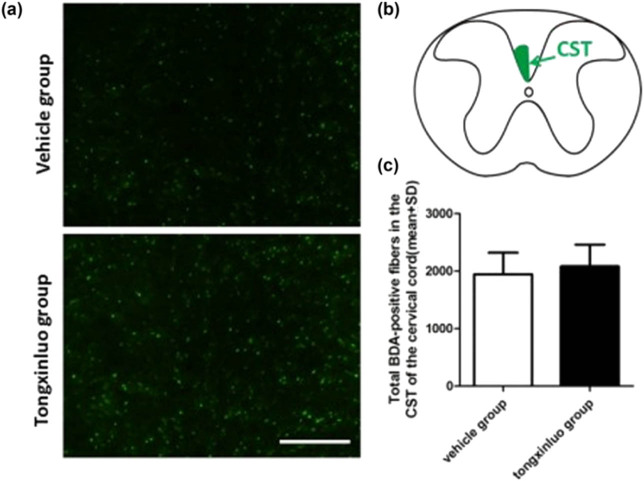



We found out that there is a high degree of overlap between the two images in Figure 5a. We checked the data, confirmed that we had misused the images, both of which actually belong to the vehicle group. However, there are no significant differences in the quantification of spinal white matter axonal density between rats treated with the vehicle or tongxinluo, indicating a negative result (Figure 5c). Although we misused the images, it did not affect our final results and conclusion.

We would like to replace the erroneous image and can guarantee the authenticity and reliability of the data. Furthermore, as the image of the vehicle group in Figure 5a appears darker than the previously provided image, we would like to provide the original image again for replacement. The following corrections have been made.

Thank you for providing us with the opportunity to make corrections. The authors sincerely apologize for any inconvenience caused.

**Correct figures:** This is the final version of Figure 5 that we would like to present.



